# Depression and associated factors in Brazilian women according to Vigitel, 2023

**DOI:** 10.1590/1806-9282.20251095

**Published:** 2026-04-20

**Authors:** Maria Eduarda Selbach Pertile, Erika Helena Konrath Hernandes, Marina de Oliveira de Vasconcellos Luiz, Augusto Kenzo Colombo Ikeda, Camile Ramos Ribeiro, Jefferson Traebert, Daniela de Rossi Figueiredo

**Affiliations:** 1 University of South Santa Catarina, Pedra Branca Campus, Medical School – Palhoça (SC), Brazil.

**Keywords:** Depression, Women, Social determinants of health

## Abstract

**OBJECTIVE:**

The aim of this study was to estimate the prevalence and factors associated with depression among Brazilian women based on the 2023 Vigitel National Survey.

**METHODS:**

This descriptive and analytical study utilized data from the Vigitel National Survey conducted by the Brazilian Ministry of Health. The sample included 13,558 women aged 18 years and older. Researchers analyzed depression prevalence, defined as a dichotomous outcome, alongside sociodemographic variables (age, education level, marital status, skin color, homeownership) and other factors such as health insurance coverage, physical activity, alcohol consumption, smoking, and body mass index. The study team performed descriptive and analytical statistics and tested associations using Pearson's chi-square test with a significance level of p<0.05.

**RESULTS:**

They found a depression prevalence of 17.5% (95%CI 16.8–18.1). Higher prevalence was observed among older women, those with up to 7 years of education, and those who reported being separated or divorced. White women showed the highest prevalence of depression. Additionally, physical inactivity, daily tobacco use, and a body mass index below 18 were associated with an increased occurrence of depression.

**CONCLUSION:**

Older women, those with lower education levels, separated women, physically inactive individuals, and smokers exhibited higher depression prevalence. These findings highlight the need for targeted care strategies and interventions addressing associated risk factors.

## INTRODUCTION

Depression in women is a mental health issue influenced by biological, psychological, and social factors, characterized by persistent sadness, anhedonia, physical symptoms, anxiety, irritability, and risky behaviors^1^. In 2019, it contributed to 37.3% of years of life lost due to disability, followed by the pandemic, which led to an increase in the prevalence of cases^1^. Hormonal factors, particularly during physiological periods of fluctuation such as puberty, maternity, and the puerperium, significantly contribute to its occurrence^2^. Estrogen secretion changes influence the pathophysiology of depression, especially during climacteric^3,4^. Research conducted during the pandemic revealed a higher prevalence of depression in women (21%) compared to men (17.3%)^5^. The symptoms have twice the prevalence in the female population, which can be explained by women's increased exposure to stressors such as gender discrimination and societal pressures^6,7^. Domestic violence, fertility difficulties, pregnancy, miscarriages, and postpartum depression have also been associated with depression^8^. Black and brown women are more likely to experience depression and have less access to treatment^9,10^.

Low education levels have been identified as a contributing factor, while living with a partner, having social and family support, and engaging in regular physical exercise are protective factors against the disease^11,12^. Women who heavily consume alcohol are 2.1 times more likely to develop mental disorders compared to abstainers, while smokers are 3.5 times more likely^13^.

Considering the increased prevalence of depression in women following the Covid-19 pandemic, and the consequences of disabilities caused by mental disorders, it becomes crucial to recognize the social determinants and risk factors associated with depression in the Brazilian female population. This knowledge is essential to support intervention and prevention strategies. Therefore, this study aimed to estimate the factors related to depression in Brazilian women, based on data from the 2023 Vigitel National Survey.

## METHODS

This is a descriptive and analytical study based on secondary data from the Vigitel National Survey, conducted by the Health Surveillance Secretariat (SVS) of the Brazilian Ministry of Health, which aims to monitor the frequency and distribution of risk and protective factors for non-communicable chronic diseases in the Brazilian population through telephone interviews across the 26 state capitals of Brazil. The study team analyzed data from the year 2023. The sample consisted of women aged 18 years or older, totaling 13,558 participants.

The variables analyzed included outcome—prevalence of depression (yes/no) and exploratory variables: sociodemographic factors such as age (categorized as 18–24, 25–34, 35–44, 45–54, 55–64, and 65 years or older), schooling (categorized as no formal education, 1–7 years, 8–11 years, 12 years or more), marital status (single, married, separated/divorced, widowed), skin color (White, Black, Mixed-race, Asian, Indigenous), and homeownership (yes/no). The researchers assessed healthcare access through private health insurance ownership (yes/no) and included health-related factors such as physical activity (yes/no), alcohol consumption (yes/no), smoking (yes/no), and Body Mass Index (underweight, normal weight, overweight, obesity).

The study team performed descriptive and analytical statistical analyses and tested associations between the outcome and exploratory variables using Pearson's chi-square test in Stata® software, version 13.

## RESULTS

The results indicated a prevalence of depression of 17.5% (95%CI 16.8–18.1). According to the descriptive analysis of the sample, the most prevalent age group among women was 25–34 years (44%). Additionally, 44% had 8–11 years of education, 34% reported being legally married, and 33% were single. The most common skin color in the sample was brown (47%). Physical exercise was reported by 54% of women, while 7% had a smoking habit, 45% reported not consuming alcohol, 65% were at least overweight, and 56% reported not having health insurance (Table 1).

**Table 1:** Prevalence of depression, sociodemographic variables, lifestyle, and health condition among Brazilian women aged 18 and over, according to Vigitel, 2023 (n=13,558).

Variables		n	%	95%CI
Age (years) (n=13,558)				
	18–24	2,856	21.07	20.4; 21.7
	24–34	5,912	64.67	42.8; 44.4
	35–59	4,001	29.51	28.7; 30.3
	>60	789	5.82	5.4; 6.2
Schooling (years) (n=13,558)				
	0–7	2,738	20.19	19.5; 20.9
	8–11	5,955	43.92	43.2; 44.7
	>12	4,865	35.88	35.1; 36.7
Marital status (n=13,492)				
	Single	4,488	33.26	32.5; 34.1
	Legally married	4,557	33.78	33.0; 34.6
	Stable union (>6 months)	1,480	10.97	10.4; 11.5
Widowed		1,642	12.17	11.6; 12.7
	Separated or divorced	1,325	9.83	9.3; 10.3
Skin color (n=13,412)				
	White	5,044	37.61	36.8; 38.4
	Black	1,849	13.79	13.2; 14.4
	Brown	6,242	46.54	45.7; 47.4
	Indigenous/Asian	277	2.07	1.8; 2.3
Health insurance coverage (n=13,536)				
	Yes	5,952	43.97	43.1; 44.8
	No	7,584	56.03	55.2; 56.9
Homeownership (n=13,547)				
	Yes	5,171	38.17	37.3; 39
	No	8,376	61.83	61; 62.6
Alcohol consumption (n=13,555)				
	Yes	4,633	34.26	33.4; 35.1
	No	6,182	45.61	44.8; 46.4
	Prefer not to answer	2,729	20.13	19.5; 20.8
Physical activity (n=13,558)				
	Yes	7279	53.69	52.8; 54.5
	No	6,279	46.31	45.5; 47.1
Smoking (n=13,558)				
	Daily smoker	733	5.41	5.0; 5.8
	Occasional smoker	188	1.39	1.2; 1.6
	Non-smoker	12,637	93.21	92.8; 93.6
BMI (kg/m^2^) (n=12,168)				
	<18	321	2.64	2.4; 3.0
	18–24	3.983	32.73	32.0; 33.6
	≥25	7.864	64.63	63.8; 65.5

CI: confidence interval; BMI: body mass index.

The study observed a significant age-related increase, with a higher prevalence among older adult women (19.3%; 95%CI 18.1–20.6) and young adult women (18.3%; 95%CI 17.3–19.3) compared to younger individuals. Depressive symptoms were more frequent among women with up to 7 years of schooling (20.1%; 95%CI 18.7–21.7). The study found a greater prevalence among participants who reported being separated or divorced (22%; 95%CI 20.2–24.7). White women exhibited the highest rate of depression (19%; 95%CI 17.9–20.1). Physical inactivity was associated with a higher occurrence of depression (19.7%; 95%CI 18.7–20.7). Women who reported daily tobacco use had a higher prevalence of depression (27.3%; 95%CI 24.2–30.6) compared to their counterparts. The study also observed a greater prevalence among women with a BMI below 18 (20.2%; 95%CI 16.2–25.0). On the other hand, variables such as health insurance coverage, alcohol consumption, and homeownership did not show a significant association with the presence of depression (Table 2).

**Table 2:** Association between depression prevalence and sociodemographic variables, lifestyle, and health condition among Brazilian women aged 18 and over, according to Vigitel, 2023 (n=13,558).

Variables		Depression		No depression		p-value*
		n(%)	95%CI	n(%)	95%CI	
Age (years) (n=13,558)						<0.001
	18–24	392 (13.7)	12.5; 15.0	2.464 (86.3)	85.0; 87.5	
	24–34	1.080 (18.3)	17.3; 19.3	4.832 (81.7)	80.7; 82.7	
	35–59	773 (19.3)	18.1; 20.6	3.228 (80.7)	79.4; 81.9	
	>60	123 (15.6)	13.2; 18.3	666 (84.4)	81.7; 86.8	
Schooling (years) (n=13,558)						<0.001
	0–7	551 (20.1)	18.7; 21.7	2.187 (80)	78.3; 81.3	
	8–11	1.012 (17)	16.0; 18.0	4.943 (83)	82.0; 84.0	
	>12	805 (16.5)	15.5; 17.6	4.060 (83.5)	82.4; 84.5	
Marital status (n=13,492)						<0.001
	Single	739 (16.5)	15.4; 17.6	3.749 (83.5)	82.4; 84.6	
	Legally married	751 (16.5)	15.4; 17.6	3.806 (83.5)	82.4; 84.6	
	Stable union (>6 months)	1.218 (82.3)	15.8; 19.7	262 (17.7)	80.3; 84.1	
	Widowed	1.325 (80.7)	17.4; 21.2	317 (19.3)	78.7; 82.5	
	Separated or divorced	1.029 (77.7)	20.2; 24.7	269 (22.3)	75.5; 79.8	
Skin color (n=13,412)						0.002
	White	959 (19)	17.9; 20.1	4.085 (80.1)	79.9; 82.0	
	Black	293 (15.6)	14.2; 17.6	1.556 (84.1)	82.4; 85.7	
	Brown	1,040 (16.7)	15.7; 17.6	5.202 (83.3)	82.4; 84.2	
	Indigenous/Asian	52 (18.8)	14.5; 23.8	225 (81.2)	76.2; 85.4	
Health insurance coverage (n=13,536)						0.099
	Yes	1,077 (18)	17.1; 19	4,875 (82)	80.9; 82.8	
	No	1,290 (17)	16.1; 17.8	6,294 (83)	82.1; 83.8	
Homeownership (n=13,547)						0.119
	Yes	870 (16.8)	15.8; 17.8	4,301 (83.2)	82.1; 84.1	
	No	1,497 (17.9)	17; 18.7	6,879 (82.1)	81.3; 83	
Alcohol consumption (n=13,555)						0.622
	Yes	792 (17)	16; 18.1	3,852 (83)	81.8; 84.0	
	No	1,088 (17.6)	16.6; 18.6	5,094 (82.4)	81.4; 83.3	
	Prefer not to answer	488 (17.9)	16.5; 19.4	2,241 (82.1)	80.6; 83.5	
Physical activity (n=13,558)						<0.001
	Yes	1.132 (15.5)	14.7; 16.4	6.147 (84.4)	83.6; 85.3	
	No	1.236 (19.7)	18.7; 20.7	5.043 (80.3)	79.3; 81.3	
Smoking (n=13,558)						<0.001
	Daily smoker	200 (27.3)	24.2; 30.6	533 (72.7)	69.4; 75.8	
	Occasional smoker	37 (19.7)	14.6; 26.0	151 (80.3)	74.0; 85.4	
	Non-smoker	2.131 (17.5)	16.2; 17.5	10.506 (82.5)	82.5; 83.8	
BMI (kg/m^2^) (n=12,168)						<0.001
	<18	65 (20.2)	16.2; 25.0	256 (79.7)	75.0; 83.8	
	18–24	615 (15.4)	14.3; 16.6	3.368 (84.6)	83.4; 85.6	
	≥25	1.481 (18.8)	18.0; 19.7	6.383 (81.2)	80.3; 82.0	

* Pearson's chi-square test. CI: confidence interval; BMI: body mass index.

## DISCUSSION

This study identified a prevalence of depression in 17.5% of Brazilian women, with a significant increase with age. It was associated with factors such as low schooling level, being separated or divorced, being White, and having a BMI below 18. Additionally, physical inactivity and daily tobacco use emerged as significant behavioral factors, with daily tobacco use being most strongly associated with depression.

Older women up to 79 years old exhibited a higher prevalence of depression. Biological changes during climacteric and menopause represent a risk factor for mental health^14^. A decrease in estrogen levels, related to ovarian aging and the transition to a non-reproductive life, is associated with depression, anxiety, and mild cognitive complaints due to the hormone's role in neural plasticity and neuroprotection^14^. A study with 58 women aged 35–56 in the early stages of menopause revealed that 70% developed mood alterations, indicating that endocrine changes affect the ability to cope with stressors and exacerbate depressive symptoms^15^. The symptomatology can also worsen depressive signs, hot flashes, and sleep disturbances^14^.

Regarding marital status, separated or divorced women showed a higher prevalence of depression^11,16^. This finding aligns with previous research indicating that living with a partner and having social support play a protective role against the development of depression. Women without a partner have a 74% higher risk of developing depression^17^.

Regarding education, the literature well supports the relationship with depression; individuals with lower schooling levels have a higher prevalence of the condition. Women with education below high school have 3.5 times the chance of developing depression^17^. Authors infer that this limitation affects autonomy and participation in social interactions and is associated with worse socioeconomic conditions, such as unemployment, poor housing, and inadequate nutrition^11,17,18^.

In this study, self-declared White women showed a higher prevalence of depression, although most participants in the sample identified as mixed-race. This finding contradicts studies suggesting a higher prevalence of depression among non-White individuals, with Black women being most affected, constituting 52.8% of cases^11,19^. Authors suggest that Black women, often subject to fewer educational, financial, and social opportunities, face conditions that affect the health-illness process. This vulnerability becomes even more evident among Black women living in extreme poverty or outside the formal labor market, which increases the risk of developing depression^20^.

Daily tobacco use was significantly associated with depression. A meta-analysis revealed a 50% increase in the likelihood of depression among smokers in cross-sectional studies and a 62% higher risk of developing the disease in longitudinal research^16^. Smoking is associated with a higher risk of onset and worsening of depressive episodes, as nicotine alters brain neurotransmitter levels^21^. These chemical changes affect emotional stability, creating a cycle of temporary relief but exacerbating symptoms in the long term^22^. Smokers with depression tend to consume more tobacco, have higher dependence, and experience intense withdrawal^23^.

Furthermore, the higher prevalence of depression among sedentary women (19%) confirms the strong association between physical inactivity and depressive states. In contrast, regular physical activity offers psychophysiological benefits and is effective in both preventing and treating the disease^11,17,24^.

Women with a BMI lower than 18 kg/m^2^ experienced depressive episodes, which may be related to inadequate nutritional conditions that affect brain function and increase the risk of depression, along with the bidirectional relationship between depression and loss of appetite and weight. Contrary to these findings, the literature points to a higher prevalence of depression among obese individuals^24^.

Some limitations of this study arise from its cross-sectional design, which does not allow for causal inferences. Due to the self-reported nature of the data, the information may be subject to bias. Thus, we should interpret the results with caution. However, it is noteworthy that the study team conducted the research with a representative sample of Brazilian women, making the results reliable for national estimates.

## CONCLUSION

Social determinants such as age, education level, health behaviors, physical inactivity, and smoking were identified as associated risk factors. These findings highlight the need for targeted interventions for more vulnerable groups to reduce mental health disparities, as well as to enable the development of more effective prevention strategies and improve the quality of life for Brazilian women.

## Data Availability

The datasets generated and/or analyzed during the current study are available from the corresponding author upon reasonable request.
